# Multiscale Risk Factors of Cardiovascular Disease: CLSA Analysis of Genetic and Psychosocial Factors

**DOI:** 10.3389/fcvm.2021.599671

**Published:** 2021-03-16

**Authors:** Gabriella Menniti, Catherine Paquet, Hannah Yang Han, Laurette Dube, Daiva E. Nielsen

**Affiliations:** ^1^School of Human Nutrition, McGill University, Montreal, QC, Canada; ^2^Faculté des Sciences Administratives, Université Laval, Québec, QC, Canada; ^3^Australian Centre for Precision Health, University of South Australia, Adelaide, SA, Australia; ^4^McGill Center for the Convergence of Health and Economics, Desautels Faculty of Management, Montreal, QC, Canada

**Keywords:** cardiovascular disease, polygenic risk score, depression—epidemiology, social isolation, aging

## Abstract

**Background:** Cardiovascular disease (CVD) is a complex disease resulting from multiscale risk factors including genetics, age, and psychosocial factors (PSFs) such as depression and social isolation. However, previous research has lacked in operationalizing multiscale risk factors to determine individual and interactive associations over the life course. Therefore, this study aimed to evaluate individual and interactive associations of multiscale risk factors for CVD outcomes including genetics and PSFs at middle and older-aged stages of the life course.

**Methods:** Baseline data from the Canadian Longitudinal Study on Aging (CLSA; *n* = 9,892 with genome-wide genotyping data) was used for this investigation. A 39 single nucleotide polymorphism polygenic risk score (PRS) for CVD was constructed. PSFs consisted of: (1) Depressive symptoms categorized into: “none” (Group 1, reference), “current” (Group 2), “clinical depression with no current symptoms” (Group 3), and “potential, recurrent depression” (Group 4); and (2) Social isolation index as a binary variable comprised of marital status, living arrangements, retirement status, contacts, and social participation. Heart-related disorders (HRD: myocardial infarction, angina and heart disease) was the primary outcome of interest and peripheral/vascular-related disorders (PVRD: stroke, peripheral vascular disease and hypertension) was the secondary outcome. Multivariable logistic regression models adjusted for socio-demographic factors were conducted stratified by age group (middle-aged: 45–69 years, older-aged: ≥70 years).

**Results:** PRS was associated with HRD among middle- and older-aged participants [OR (95% confidence interval)] [1.06 (1.03–1.08), 1.06 (1.03–1.08), respectively]. Most depressive symptoms groups compared to the reference associated with HRD and PVRD, but only Group 4 associated with PVRD among older-aged [1.69 (1.08–2.64)]. Social isolation was associated with only PVRD among middle-aged [1.84 (1.04–3.26)]; however, socially isolated CLSA participants were underrepresented in the genotyped cohort (1.2%). No significant PRS^*^PSFs interactions were observed.

**Conclusions:** Genetics and PSFs are independently associated with CVD. Varying observations across age groups underscores the need to advance research on multiscale risk factors operating both at a given point in time and over the life course. Future cohort studies may benefit from use of mobile assessment units to enable better reach to socially isolated participants for collection of biospecimens.

## Introduction

Cardiovascular disease (CVD) is the leading cause of death worldwide, accounting for nearly 30% of deaths globally, and the second leading cause of death in Canada (1, 2). CVD can be categorized into heart-related disorders (HRD) including myocardial infarction, angina and heart disease and peripheral/vascular-related disorders (PVRD) including cerebrovascular accident (CVA), hypertension and peripheral vascular disease (3). Multiscale risk factors consider combinations of factors that are involved along the pathway of disease pathogenesis and represent a more all encompassing approach to studying disease risk (4). This investigation assessed the associations between CVD and multiscale risk factors of genetics, psychosocial factors (PSFs) and life course stage.

Genome-wide association studies (GWAS) have identified several single nucleotide polymorphisms (SNPs) that are associated with an increased risk of coronary artery disease (CAD), the most common form of heart disease (5). From these, a polygenic risk score (PRS) has been developed to quantify genetic risk according to the number of SNP alleles carried by an individual (6). GWAS have primarily focused on SNPs associating with HRD (6) and to a lesser extent with PVRD (7), and the genetic predisposition may differ between types of CVD (8). While GWAS with CVD outcomes have predominantly been conducted among cohorts of middle-aged individuals, emerging evidence suggests that effects of genetic variation on health outcomes may dampen as individuals approach the later stages of the life course (9). Non-genetic risk factors may be increasingly relevant for chronic disease risk or co-morbidities at the later stages of life as experience and environmental exposure cumulate over a person's life course (9, 10), underscoring the importance of assessing the performance of PRS in older-aged groups. Moreover, recent gene-environment interaction studies provide supportive evidence that non-genetic factors (healthy lifestyle) may counteract polygenic risk of CAD and may be particularly beneficial for subgroups at highest polygenic risk (6). While lifestyle is an important modifiable risk factor, no previous study has examined psychosocial factors (PSFs) as possible moderators of polygenic risk of CAD despite robust evidence for individual associations between PSFs and CVD outcomes. In particular, depression has been consistently associated with CVD (3, 11), while social isolation is a growing problem among older-aged individuals and has been more recently linked with CVD (12, 13). While they can be related constructs, social isolation does not consistently correlate with depression (14) as it more often reflects a circumstantial rather than emotional state (such as loneliness, a strong risk factor for depression) (15). Poor PSFs can negatively influence lifestyle, including being linked with a less healthful diet and reduced physical activity (16–18). Given the growing recognition of the role of psychosocial well-being in chronic disease risk (3), PSFs warrant consideration as possible moderators of the associations between genetic susceptibility to CVD and CVD outcomes, as they represent environmental contributors to CVD that are upstream of lifestyle which has been reported to be a modifier of the association between genetic susceptibility to CVD and CVD outcomes (6). Therefore, the objective of the present study was to assess individual and interactive associations of PSFs (depression and social isolation) and polygenic risk of CAD with CVD outcomes among middle-aged and older adults. Since depression and social isolation do not necessarily manifest together, we evaluated each PSF as a separate exposure variable.

## Methods

### Study Design and Cohort

This cross-sectional analysis used baseline data from the Canadian Longitudinal Study on Aging (CLSA). Data from 50,000 participants between the ages of 45–85 years old (forming the CLSA Tracking and Comprehensive Cohorts) upon recruitment was collected *via* telephone survey on demographic, behavioral, physical, economic, psychological, and social aspects of their lives (19). The Comprehensive Cohort is a subset of 30,000 CLSA participants that had in-depth information collected at data collection sites (DCS) including further interviews, in-person physical examinations and biospecimen sample collections. Out of the CLSA Comprehensive Cohort, 9,896 randomly sampled participants had genome-wide genotyping performed on DNA samples (19).

### Polygenic Risk Score

Genome-wide genotyping was performed using the Affymetrix UK Biobank Axiom array (20). Out of 50 SNPs used to create a PRS by Khera et al. (6), 39 were present in the CLSA genotyping data. The PRS was made by multiplying the number of CAD risk alleles by their weighted risk estimate (natural log of published odds ratio) for each of the SNPs seen in Supplementary Table 1 (6). These values were then summed across all SNPs and multiplied by the total number of SNPs divided by the sum of the weighted risk estimates. The resulting PRS used in the present analysis could range from 0 (no SNPs) to 78 (all 39 SNPs).

### Depressive Symptoms

Depressive symptoms were assessed using the short form of the Center for Epidemiological Studies—Depression (CES-D-10) Scale. Scores ranged from 0 to 30, where a score of >10 indicated current depressive symptoms (positive screen for depression) and a score of <10 indicating no evidence of current depressive symptoms (negative screen) (21). Additionally, participants were assessed for presence of clinical depression by being asked “Has a doctor ever told you that you suffer from clinical depression?” (21). Based on these two assessments of depressive symptoms, participants were categorized into four groups as previously described by Liu et al. (3). Group 1 had no evidence of any depressive symptoms with a negative screen for depression and a negative response to the question on clinical depression. Group 2 are considered to have *current* depressive symptoms with a positive screen for depression but a negative response to the question on clinical depression. Group 3 are considered clinically depressed without any current depressive symptoms with a negative screen for depression but a positive response to the question on clinical depression. Group 4 have potential, recurrent depression with both a positive screen for depression and a positive response to the question on clinical depression.

### Social Isolation Index

Social Isolation Index (SII) was created according to methods developed by Menec et al. (22) using responses to five sets of questions in regard to marital status, living arrangements, social contacts, retirement status and social participation. The index was scored between 0 and 5 with higher scores indicating higher levels of social isolation (22). A social isolation score from 0 to 2 was classified as not socially isolated (coded as 0) and a score from 3 to 5 was classified as socially isolated (coded as 1) (22).

### Outcome Measures

The outcome measures were defined in the same manner as Liu et al. for consistency of methods (3). The six CLSA questions for physician-diagnosed CVD were subdivided into two groups: heart-related disorders (HRD) and peripheral/vascular-related disorders (PVRD). HRD was the primary outcome of interest, composed of heart disease, myocardial infarction and angina. These were each assessed with the following questions: “Has a doctor ever told you that you have… (i) heart disease (including congestive heart failure or chronic heart failure)?; (ii) a heart attack or myocardial infarction?”; (iii) angina (or chest pain due to heart disease)?” PVRD was the secondary outcome of interest and was composed of hypertension, cerebrovascular accident and peripheral vascular disease. These were each assessed with the follow questions: “Has a doctor ever told you that you have… (i) high blood pressure or hypertension?”; (ii) experienced a stroke or cerebrovascular accident (CVA)?”; (iii) peripheral vascular disease or poor circulation in your limbs?” These CVD subgroups share common risk factors and pathophysiology with the major point of convergence being anatomical location (23).

### Covariates

Age (years) and the first five principal components of ancestry were included in the regression model as continuous co-variates. The following categorical co-variates were also included: biological sex (male vs. female), education level (<secondary school; secondary school graduate but no post-secondary education; post-secondary education but below bachelor's degree; bachelor's degree; and > bachelor's degree), province at recruitment (Alberta, British Columbia, Manitoba, New Brunswick, Newfoundland and Labrador, Nova Scotia, Ontario, Prince Edward Island, Quebec, Saskatchewan), total household income ($20,000 or more, but <$50,000; $50,000 or more, but <$100,000; $100,000 or more, but <$150,000; $150,000 or more), smoking status (current smoker; former smoker; and never smoked), urban/rural classification (urban; rural; postal code link to dissemination area) and immigration status (immigrant vs. not an immigrant). A statistical model with more conservative covariate adjustment was also conducted and results are presented in the Supplementary Material.

### Multiple Imputation

A statistical multiple imputation (MI) was performed using SAS 9.4 to minimize bias due to missing data. Missing data was not substantial with the exception of one variable used to construct the SII, “social contacts with children,” which had *n* = 1,488 observations missing (15%). The fully conditional method (FCS) was used to impute missing variables since the outcome variables (HRD and PVRD) are binary (24). MI was performed using 20 imputation datasets. Results reported herein were generated with the use of MI. Corresponding results generated from a complete case analysis (CCA) are included as Supplementary Material.

### Statistical Analysis

Characteristics between the middle-aged and older-aged participants were compared using Chi square tests and independent *t*-tests. Multi-variable logistic regression models were used to test the main and interactive associations of PRS and PSFs with HRD and PVRD. Separate models were run for depression and SII. For depressive symptoms, the reference category was Group 1 (no depressive symptoms). The reference category for the social isolation variable was not socially isolated (coded as 0). These logistic regression models were performed for all participants, and also for a predefined subgroup analysis stratified by age: middle-aged participants between 45 and 69 years old (*n* = 7,155) and older participants aged 70 years and above (*n* = 2,737). This age stratification was chosen because the mean age of individuals in GWAS of CVD is most commonly 69 or younger (6), thus, age 70 represented a target cut-off to evaluate the performance of a PRS for CAD amongst an older-aged sample. All analyses were conducted using SAS version 9.4 (SAS Institute, North Carolina) and all *p*-values were two-sided with alpha set at 0.05.

As a sensitivity analysis, the continuous PRS was separated into quintiles to test the interaction association between extreme categories of PRS and the PSFs. Quintile 1 (lowest PRS scores) was compared to the reference quintile 5 (highest PRS scores) since categorizing a PRS may enable detection of associations between the extremes of genetic risk (6).

## Results

### Population Characteristics

Our analytical sample included *n* = 9,892 of the 9,896 participants who had data available for the exposure (PRS and PSFs) and outcomes variables (HRD and PVRD) upon MI. Four participants had genetic data missing for principal components analysis of ancestry. Upon PRS calculation with genotyping data for the analytical sample, the range of PRS was between 22.2–54.5. Table 1 shows the characteristics of the analytical sample based on the MI analysis. Middle-aged adults comprised 72% of the sample and 28% were older-aged adults. Compared to middle-aged groups, the older-aged group had a lower prevalence of the following characteristics: high income bracket households, non-Caucasian participants, current smokers, post-secondary education graduates, rural-residing participants, and group 3 and 4 depressive symptoms. The older-aged group had a higher prevalence of immigrants and socially isolated participants, and the mean PRS was slightly lower compared to the middle-aged group. Overall, the majority of all participants fell within Group 1 of depressive symptoms (no depressive symptoms) and were considered not socially isolated. As expected, a greater proportion of older-aged participants reported at least one CVD outcome compared to the middle-aged subgroup. These descriptive statistics were similar in the CCA (see Supplementary Table 2).

**Table 1:** Participant characteristics.

**Variable**	**Total** **(*n* = 9,892)**	**Middle-aged** **(*n* = 7,155)**	**Older-aged** **(*n* = 2,737)**	***P*-value**^**†**^
Age (years)^*^	63.0 (10.2)	57.9 (6.6)	76.3 (4.2)	<0.0001
Biological sex (male)	49.2%	48.6%	50.6%	0.0740
Total household income ($)				<0.0001
Less than $20,000	5.6%	5.2%	6.7%	
$20,000 or more, but <$50,000	23.5%	17.9%	38.2%	
$50,000 or more, but <$100,000	35.2%	34.4%	37.4%	
$100,000 or more, but <$150,000	19.8%	22.6%	12.4%	
$150,000 or more	15.9%	19.9%	5.4%	
Self-reported ethnicity				<0.0001
Caucasian	92.0%	90.9%	94.7%	
Non-Caucasian	8.0%	9.1%	5.3%	
Smoking status				<0.0001
Current smoker	9.5%	11.2%	5.0%	
Non-smoker	47.1%	48.3%	43.9%	
Former smoker	43.4%	40.5%	51.1%	
Education level				<0.0001
Less than secondary school graduate	5.5%	3.7%	10.2%	
Secondary school graduate but no post-secondary education	9.8%	9.1%	11.8%	
Post-secondary education but below a bachelor's degree	40.0%	39.8%	40.6%	
Bachelor's degree	23.1%	25.5%	16.7%	
Higher than a bachelor's degree	21.5%	21.8%	20.7%	
Province at recruitment				0.4723
Alberta	9.7%	9.7%	9.7%	
British Columbia	20.9%	20.6%	21.6%	
Manitoba	10.3%	10.3%	10.1%	
Newfoundland and Labrador	7.3%	7.4%	7.1%	
Nova Scotia	10.1%	10.2%	10.0%	
Ontario	21.3%	21.0%	22.3%	
Quebec	20.4%	20.8%	19.3%	
Urban vs. rural classification				0.0001
Rural	7.7%	8.3%	6.3%	
Urban	90.9%	90.6%	91.8%	
Not available	1.4%	1.2%	1.9%	
Immigration status				<0.0001
Immigrant	17.7%	15.7%	23.1%	
Not an immigrant	82.3%	84.3%	76.2%	
Polygenic risk score (PRS)^*^	37.7 (4.2)	37.8 (4.2)	37.6 (4.1)	0.0330
Depressive symptoms				<0.0001
Group 1: no depressive symptoms	73.2%	71.3%	78.2%	
Group 2: current depressive symptoms	10.0%	9.4%	11.4%	
Group 3: clinically depressed but without any current depressive symptoms	10.7%	12.3%	6.7%	
Group 4: potential, recurrent depression	6.1%	7.0%	3.7%	
Social isolation				0.0015
Not socially isolated	98.8%	99.0%	98.2%	
Socially isolated	1.2%	1.0%	1.8%	
Heart-related disorders (HRD)				<0.0001
At least one of the heart-related disorders	13.0%	8.8%	24.1%	
None of the heart-related disorders	87.0%	91.2%	75.8%	
Peripheral/vascular-related disorders (PVRD)				<0.0001
At least one of the peripheral/vascular-related disorders	40.0%	33.6%	56.7%	
None of the peripheral/vascular-related disorders	60.0%	66.4%	43.3%	

† *Middle-aged vs. older-aged characteristic comparison*.

* *Cells indicate mean (standard deviation)*.

### Main Effect Associations Between PRS and CVD Outcomes

The PRS was significantly associated with HRD in the total sample as well as in the middle-aged and older-aged subgroups. The association between the PRS and PVRD was significant among the total sample and middle-aged subgroups, but was not significant in the older-aged subgroup (Table 2).

**Table 2:** Main effect associations between PRS and CVDs.

	**HRD**				**PVRD**			
**Polygenic risk score**	**OR**	**95% CI**		***P*-value**	**OR**	**95% CI**		***P*-value**
Total (*n* = 9,892)	1.06	1.04	1.07	<0.0001	1.01	1.00	1.02	0.0127
Middle-aged (*n* = 7,155)	1.06	1.03	1.08	<0.0001	1.02	1.00	1.03	0.0127
Older-aged (*n* = 2,737)	1.06	1.03	1.08	<0.0001	1.01	0.99	1.02	0.6312

### Main Effect Associations Between PSFs and CVD Outcomes

Most depressive symptoms compared to the reference (Group 1, no depressive symptoms) significantly associated with both HRD and PVRD among the total sample and middle-aged, however, only Group 4 (potential, recurrent depression) compared to the reference was significantly associated with PVRD among older-aged (Table 3). Being socially isolated compared to not socially isolated was not significantly associated with HRD among the total sample as well as the middle-aged and older-aged subgroups. Being socially isolated was significantly associated with PVRD among the total sample and middle-aged participants, but not among the older-aged subgroup (Table 4).

**Table 3:** Main effects associations between depressive symptoms and CVDs.

		**HRD**				**PVRD**			
	Depressive symptoms^*^	**OR**	**95% CI**		***P*-value**	**OR**	**95% CI**		***P*-value**
Total (*n* = 9,892)	1	1.00				1.00			
	2	1.01	0.82	1.26	0.9001	1.22	1.05	1.40	0.008
	3	1.41	1.15	1.75	0.0013	1.42	1.23	1.63	<0.0001
	4	1.57	1.57	2.04	0.0008	1.68	1.40	2.02	<0.0001
Middle-aged (*n* = 7,155)	1	1.00				1.00			
	2	1.10	0.82	1.49	0.5218	1.28	1.07	1.53	0.0058
	3	1.21	1.21	2.01	0.0006	1.49	1.28	1.74	<0.0001
	4	1.75	1.28	2.39	0.0005	1.73	1.41	2.12	<0.0001
Older-aged (*n* = 2,737)	1	1.00				1.00			
	2	0.96	0.71	1.31	0.8039	1.13	0.88	1.46	0.3275
	3	1.11	0.76	1.62	0.5920	1.17	0.85	1.61	0.3264
	4	1.26	0.78	2.04	0.3488	1.69	1.08	2.64	0.0226

* *Each group of depressive symptoms is compared to the reference (Group 1)*.

**Table 4:** Main effects associations between social isolation and CVDs.

	**HRD**				**PVRD**			
Social isolation (1 vs. 0)^*^	**OR**	**95% CI**		***P*-value**	**OR**	**95% CI**		***P*-value**
Total (*n* = 9,892)	0.98	0.58	1.65	0.9352	1.65	1.07	2.56	0.0251
Middle-aged (*n* = 7,155)	1.59	0.82	3.10	0.1738	1.84	1.04	3.26	0.0361
Older-aged (*n* = 2,737)	0.58	0.26	1.30	0.1828	1.41	0.70	2.83	0.3362

* *Those who are socially isolated (coded as 1) are compared to the reference not socially isolated (coded as 0)*.

### PRS^*^PSF Interaction Associations With CVD Outcomes

When PRS was modeled as a continuous variable, there were no significant interactions between the PRS and either PSF (depressive symptoms and social isolation) among the total sample or age subgroups. Additionally, no significant interactions or trends were observed between the extreme quintiles of PRS for either PRS^*^depressive symptoms or PRS^*^social isolation among the total analytical sample (Table 5), middle-aged or older-aged participants (results not shown). Corresponding results in the CCA were very similar to the analysis using MI (Supplementary Tables 3–7).

**Table 5:** Interaction associations between PRS and PSFs on CVDs.

		**HRD**				**PVRD**			
	PRS^*^PSFs	**OR**	**95% CI**		***P*-value**	**OR**	**95% CI**		***P*-value**
**Continuous PRS**									
	PRS^*^Dep 1	1.00				1.00			
Total (*n* = 9,892)	PRS^*^Dep 2^+^	1.04	0.98	1.09	0.1789	0.97	0.93	1.00	0.0577
	PRS^*^Dep 3	1.00	0.95	1.05	0.9242	1.01	0.98	1.04	0.5683
	PRS^*^Dep 4	0.99	0.93	1.06	0.8366	1.00	0.95	1.04	0.8926
	PRS^*^Dep 1	1.00				1.00			
Middle-aged (*n* = 7,155)	PRS^*^Dep 2	1.05	0.97	1.13	0.2299	0.97	0.93	1.02	0.2142
	PRS^*^Dep 3	0.99	0.94	1.06	0.8243	1.01	0.98	1.05	0.5429
	PRS^*^Dep 4	0.76	0.90	1.05	0.4706	1.00	0.95	1.05	0.9455
	PRS^*^Dep 1	1.00				1.00			
Older-aged (*n* = 2,737)	PRS^*^Dep 2	1.03	0.95	1.11	0.4717	1.13	0.90	1.02	0.1380
	PRS^*^Dep 3	1.01	0.92	1.10	0.8558	1.17	0.93	1.07	0.9899
	PRS^*^Dep 4	1.04	0.92	1.18	0.5047	1.69	0.87	1.08	0.5925
Total (*n* = 9,892)	PRS^*^SII 1^′^	1.06	0.93	1.20	0.3732	1.08	0.97	1.21	0.1691
Middle-aged (*n* = 7,155)	PRS^*^SII 1	1.04	0.87	1.24	0.6661	1.13	0.96	1.33	0.1304
Older-aged (*n* = 2,737)	PRS^*^SII 1	1.07	0.89	1.27	0.4736	1.03	0.89	1.20	0.6600
**Extreme quintiles of PRS (PRS 1 vs. 5)**^**$**^									
	PRS 1^*^Dep 1	1.00				1.00			
Total (*n* = 9,892)	PRS 1^*^Dep 2	0.76	0.37	1.56	0.4535	1.27	0.81	2.00	0.3025
	PRS 1^*^Dep 3	1.01	0.53	1.95	0.9693	0.91	0.59	1.40	0.6584
	PRS 1^*^Dep 4	1.43	0.56	3.64	0.4566	1.03	0.58	1.83	0.9154
Total (*n* = 9,892)	PRS 1^*^SII 1	0.53	0.09	3.35	0.5029	0.47	0.11	2.04	0.3124

+ *Dep is the depressive symptoms group that is compared to reference (group 1)*.

′ *Social isolation index (SII) compares those who are socially isolated (coded as 1) to the reference not socially isolated (coded as 0)*.

$ *PRS quintile 1 (lowest scores of PRS) is compared to the reference PRS quintile 5 (highest scores of PRS)*.

## Discussion

This observational study investigated main and interactive associations of polygenic risk and PSFs on CVD outcomes by age group using baseline data from a Canadian cohort. This represented a multiscale approach that assessed combinations of risk factors with CVD outcomes, and to our knowledge is the first such investigation evaluating genetic, psychosocial, and biological (age) risk factors with CVD.

The findings suggest that polygenic risk and PSFs are independently associated with CVD outcomes, but that these relationships may differ according to stage of the life course. GWAS have increasingly implicated common SNPs as risk factors for CVD, however, GWAS cohorts that evaluated CVD outcomes have been predominantly restricted to middle-aged adults (5). In order for our investigation to extend upon previous work on depression and CVD using CLSA data, CVD outcomes were grouped into HRD and PVRD. While our results suggest that a PRS for CAD is associated with HRD to a similar extent among both middle-aged and older-aged individuals, the association was attenuated for PVRD among the older-aged group. This may be due to the fact that the PRS is predictive of CAD and some participants with HRD were present in the PVRD group due to the nature of the construction of CVD groupings. Thus, the results for the PRS and PVRD associations should be interpreted cautiously, particularly because the associations among the total and middle-aged group were weakly significant and survival bias may have affected the outcome amongst the older-aged group. Nevertheless, future studies are warranted to evaluate the performance of PRS among different age groups, because a growing body of evidence suggests that the influence of genetic variation may differ across the life course (9). No significant interactions were observed between polygenic risk and PSFs, however, further research is required to confirm this finding, due to the limited number of participants who were socially isolated in our sample. In addition, future research should consider characterizing depression with more precision, particularly typical vs. atypical depression rather than solely detecting its presence (i.e., symptoms and clinical history) (25).

Among PSFs, various degrees of depressive symptoms were significantly associated with HRD and PVRD among middle-aged adults, but most depressive groups were not associated with CVD outcomes among older-aged adults. These findings align with previous results for depression and CVD in the larger CLSA Comprehensive Cohort (3) and suggest that depressive symptoms may play a greater role at the mid-stage of the life course. Indeed, considering stage of the life course is important because older-aged adults have been described to exhibit differential stability of emotional experience such that positive states are maintained longer, and negative states are more quickly dismissed (26). This aging-related motivational shift may explain why depressive symptoms were not associated with CVD outcomes among the older-aged subgroup to the same degree that we observed among the middle-aged subgroup.

Social isolation was significantly associated with increased risk of PVRD among the middle-aged subgroup, but not the older-aged subgroup. This finding was unexpected since elderly adults have been identified as more vulnerable to social isolation (12). However, a likely explanation for our finding is an under-representation of socially isolated CLSA participants in the subset that had genome-wide genotyping performed, which may have impacted statistical power particularly among the elderly subgroup of our analysis that had a very low prevalence of social isolation. Menec et al. recently reported the prevalence of social isolation in the overall CLSA (*n* = 47,752) to be 5.1% (22). The prevalence in our analytical sample, comprised of the participants who had genotyping data available, was remarkably lower at 1.2%. The Comprehensive Cohort was chosen based on proximity and ability to be present at a CLSA data collection site (DCS) where blood samples were collected. Participants who were unable or unwilling to go to a DCS were excluded (27). CLSA participants who are socially isolated likely could not visit a DCS due to access barriers (e.g., living outside of proximity buffer or not being able to access transportation). CLSA follow-up efforts may benefit from incorporating mobile examination centers so that physical assessments and biospecimen collection can be obtained from a greater proportion of socially isolated participants, particularly because social isolation may be an emerging risk factor for CVD (12). Indeed, mobile examination centers have been used successfully in other population cohort studies to better reach participants who are unable or unwilling to go to a DCS (28).

Strengths of this investigation include the use of a national cohort with detailed data on participant genetics, health and lifestyle, and psychosocial factors. Additionally, we applied methods that were used in previous related studies for consistency, including our construction of a PRS for CAD (6) and our use of the same CVD outcomes (3), depressive symptoms (3), and SII (22) groupings that were utilized by separate investigations conducted with CLSA data. Since CVD affects men and women differently, we performed a supplementary sex-stratified analysis (data not shown). Our results were largely unaltered, with the exception that the main effect association between social isolation and PVRD was only statistically significant among men. However, given the low prevalence of socially isolated participants, this observation should be interpreted with caution.

A number of limitations to our approach must be acknowledged. We did not assess other relevant PSFs, such as anxiety (which was not assessed in the CLSA) and social support. In addition, the non-specific format of CLSA questions about participant CVD diagnosis history could have introduced response bias (e.g., only providing a response option for heart disease rather than specifying coronary or congenital heart disease). To account for this measurement error, CVD variables were categorized into broad groupings rather than individual CVD outcomes (3). In addition, the use of reported physician diagnosed CVD, rather than direct confirmation of disease, is a limitation. Potential survival bias in the CLSA cohort may have prevented certain associations from being detected. For example, if mortality from CVD is truly higher among older individuals with less favorable genetic or psychosocial status, elderly participants who have been diagnosed with CVD and have high polygenic risk, or are socially isolated or present with depression, would be underrepresented in CLSA (29). We did not correct for multiple testing; however, a Bonferroni correction may not be appropriate owing to the possible interrelationship between PSFs. Moreover, due to the cross-sectional nature of the study the directionality of associations between PSFs [particularly depression (30)] and CVD outcomes cannot be determined, and no data on further incidence of CVD according to PSFs or PRS was available at the time of this analysis. Lastly, lack of genetic data for a larger sample of socially isolated participants may have also affected statistical power to detect certain main-effect or interactive associations.

This study provides evidence that polygenic risk of CAD and PSFs are associated with CVD outcomes, but that stage of the life course and type of CVD are important considerations underscoring the importance of a multiscale risk factor approach. Future studies that explore the bidirectional nature of depression and CVD and identification of mediators of associations between PSFs and CVD, potentially lifestyle, are warranted in order to provide insight into multiscale strategies to prevent CVD.

## Data Availability Statement

The datasets presented in this article are not readily available because data are available from the Canadian Longitudinal Study on Aging (www.clsa-elcv.ca) for researchers who meet the criteria for access to de-identified CLSA data. Requests to access the datasets should be directed to www.clsa-elcv.ca.

## Ethics Statement

The studies involving human participants were reviewed and approved by McGill University Faculty of Agricultural and Environmental Sciences Research Ethics Board. The patients/participants provided their written informed consent to participate in this study.

## Author Contributions

GM conducted the statistical analysis, wrote the original draft of the manuscript, and prepared all figures and tables. CP and LD provided feedback on statistical analysis and methodology and helped review and edit the manuscript. HH assisted with statistical analyses and methodology and helped review and edit the manuscript. DN obtained ethics approval, provided access to the data, designed the statistical approach, and critically revised the manuscript. All authors contributed to the article and approved the submitted version.

## Conflict of Interest

The authors declare that the research was conducted in the absence of any commercial or financial relationships that could be construed as a potential conflict of interest.
