# Supplementary material for: Multiscale Risk Factors of Cardiovascular Disease: CLSA Analysis of Genetic and Psychosocial Factors
Source: Front Cardiovasc Med. 2021 Mar 16;8:599671. doi: 10.3389/fcvm.2021.599671 (PMC8007777; doi:10.3389/fcvm.2021.599671)
Supplement: Supplementary file 1 [file Data_Sheet_1.docx]

**Supplementary Tables**

**Table S1 Single Nucleotide Polymorphisms used to create Polygenic Risk Score for Coronary Artery Disease (n=39)**

| **Gene Locus** | **SNP** | **Ln (Published  Odds Ratio)** |
| --- | --- | --- |
| *SORT1* | rs599839 | 0.104 |
| *PPAP2B* | rs17114036 | 0.104 |
| *PCKS9* | rs11206510 | 0.077 |
| *IL6R* | rs4845625 | 0.039 |
| *MIA3* | rs17465637 | 0.131 |
| *GGCX/VAMP8* | rs1561198 | 0.049 |
| *ABCG8* | rs6544713 | 0.058 |
| *APOB* | rs515135 | 0.077 |
| *ZEB2-AC074093.1* | rs2252641 | 0.039 |
| *WDR12* | rs6725887 | 0.113 |
| *MRAS* | rs9818870 | 0.068 |
| *SLC22A4/SLC22A5* | rs273909 | 0.086 |
| *KCNK5* | rs10947789 | 0.058 |
| *ANKS1A* | rs17609940 | 0.068 |
| *PHACTR1* | rs12526453 | 0.095 |
| *SLC22A3/LPAL2/LPA* | rs2048327 | 0.058 |
| *LPA* | rs3798220 | 0.412 |
| *HDAC9* | rs2023938 | 0.068 |
| *BCAP29* | rs10953541 | 0.077 |
| *ZC3HC1* | rs11556924 | 0.086 |
| *TRIB1* | rs2954029 | 0.039 |
| *CDKN2BAS* | rs3217992 | 0.148 |
| *CDKN2A* | rs4977574 | 0.255 |
| *ABO* | rs579459 | 0.068 |
| *KIAA1462* | rs2505083 | 0.058 |
| *CXCL12* | rs501120 | 0.068 |
| *LIPA* | rs2246833 | 0.058 |
| *CYP17A1* | rs12413409 | 0.113 |
| *APOA5* | rs964184 | 0.122 |
| *HNF1A* | rs2259816 | 0.077 |
| *SH2B3* | rs3184504 | 0.068 |
| *FLT1* | rs9319428 | 0.049 |
| *COL4A1* | rs4773144 | 0.068 |
| *HHIPL1* | rs2895811 | 0.058 |
| *RASD1* | rs12936587 | 0.058 |
| *SMG6* | rs216172 | 0.068 |
| *UBE2Z* | rs46522 | 0.058 |
| *LDLR* | rs1122608 | 0.095 |
| *KCNE2* | rs9982601 | 0.122 |
| **Total** | 39 | 3.517 |

**Table S2** **Participant Characteristics (Complete Case Analysis)**

| **Variable** | **Total (n=9,892)** | **Middle-Aged (n=7,155)** | **Older Aged (n=2,737)** |
| --- | --- | --- | --- |
| Age | 63.0 (10.2) | 57.9 (6.6) | 76.3 (4.2) |
| Biological sex (Male) | 49.2% | 48.6% | 50.6% |
| Total Household Income ($) |  |  |  |
| Less than $20,000 | 5.5% | 5.1% | 6.7% |
| $20,000 or more, but less than $50,000 | 23.0% | 17.7% | 37.4% |
| $50,000 or more, but less than $100,000 | 35.3% | 34.4% | 37.8% |
| $100,000 or more, but less than $150,000 | 20.1% | 22.8% | 12.7% |
| $150,000 or more | 16.2% | 20.1% | 5.8% |
| Missing | n=616 | n=346 | n=270 |
| Self-Reported Ethnicity |  |  |  |
| Caucasian | 92.0% | 90.9% | 94.7% |
| Non-Caucasian | 8.0% | 9.1% | 5.3% |
| Missing | n=78 | n=61 | n=17 |
| Smoking Status |  |  |  |
| Current smoker | 9.5% | 11.2% | 5.0% |
| Non-smoker | 47.0% | 48.3% | 43.9% |
| Former smoker | 43.4% | 40.5% | 51.1% |
| Education Level |  |  |  |
| Less than secondary school graduate | 5.5% | 3.7% | 10.1% |
| Secondary school graduate but no post-secondary education | 9.8% | 9.1% | 11.8% |
| Post-secondary education but below a bachelor’s degree | 40.1% | 39.8% | 40.6% |
| Bachelor’s degree | 23.1% | 25.5% | 16.8% |
| Higher than a bachelor’s degree | 21.5% | 21.8% | 20.7% |
| Missing | n=21 | n=7 | n=14 |
| Province at recruitment |  |  |  |
| Alberta | 9.7% | 9.7% | 9.7% |
| British Columbia | 20.9% | 20.6% | 21.6% |
| Manitoba | 10.3% | 10.3% | 10.1% |
| Newfoundland and Labrador | 7.3% | 7.4% | 7.1% |
| Nova Scotia | 10.1% | 10.2% | 10.0% |
| Ontario | 21.3% | 21.0% | 22.3% |
| Quebec | 20.4% | 20.8% | 19.3% |
| Urban vs. Rural Classification |  |  |  |
| Rural | 7.7% | 8.3% | 6.3% |
| Urban | 90.9% | 90.6% | 91.8% |
| Not available | 1.4% | 1.2% | 1.9% |
| Immigration Status |  |  |  |
| Immigrant | 17.7% | 15.7% | 23.1% |
| Not an immigrant | 82.3% | 84.3% | 76.9% |
| Missing | n=2 | n=1 | n=1 |
| Polygenic Risk Score (PRS) | 37.7 (4.2) | 37.8 (4.2) | 37.6 (4.1) |
| Depressive Symptoms |  |  |  |
| Group 1: No depressive symptoms | 73.3% | 71.4% | 78.4% |
| Group 2: Current depressive symptoms | 9.9% | 9.4% | 11.4% |
| Group 3: Clinically depressed but without any current depressive symptoms | 10.8% | 12.3% | 6.7% |
| Group 4: Potential, recurrent depression | 6.0% | 6.9% | 3.6% |
| Missing | n=76 | n=36 | n=40 |
| Social Isolation |  |  |  |
| Not socially isolated | 99.2% | 99.4% | 98.7% |
| Socially isolated | 0.8% | 0.6% | 1.3% |
| Missing | n=3,924 | n=2,735 | n=1,189 |
| Heart-Related Disorders (HRD) |  |  |  |
| At least one of the heart-related disorders | 13.3% | 9.0% | 24.6% |
| None of the heart-related disorders | 86.7% | 91.0% | 75.4% |
| Missing | n=72 | n=39 | n=33 |
| Peripheral/Vascular-Related Disorders (PVRD) |  |  |  |
| At least one of the peripheral/vascular-related disorders | 40.1% | 33.7% | 56.8% |
| None of the peripheral/vascular-related disorders | 59.9% | 66.3% | 43.2% |
| Missing | n=76 | n=46 | n=30 |

*cells indicate mean (standard deviation) unless otherwise specified

|  | | | *HRD* | | | | | *PVRD* | | | | |
| --- | --- | --- | --- | --- | --- | --- | --- | --- | --- | --- | --- | --- |
| *Polygenic Risk Score* | OR | 95% CI | | | P-value |  | OR | | | 95% CI | | P-value |
| Total (n=9,188) | 1.06 | 1.04 | | 1.07 | <0.0001 | Total (n=9,187) | | | 1.01 | 1.00 | 1.02 | 0.0231 |
| Middle-Aged (n=6,766) | 1.05 | 1.03 | | 1.07 | <0.0001 | Middle-Aged (n=6,759) | | | 1.01 | 1.00 | 1.03 | 0.0306 |
| Older Aged (n=2,422) | 1.06 | 1.02 | | 1.07 | <0.0001 | Older Aged (n=2,428) | | | 1.00 | 0.99 | 1.03 | 0.5507 |

**Table S3** **Main Effect Associations Between PRS and CVDs (Complete Case Analysis)**

**Table S4 Main Effect Associations Between Depressive Symptoms and CVDs (Complete**

**Case Analysis)**

|  | |  | *HRD* | | | | *PVRD* | | | | |
| --- | --- | --- | --- | --- | --- | --- | --- | --- | --- | --- | --- |
| Depressive Symptoms* | Group | OR | 95% CI | | P-value | Group | | OR | 95% CI | | P-value |
| 1 | Total (n=9,138) | 1.00 |  |  |  | Total (n=9,137) | | 1.00 |  |  |  |
| 2 |  | 1.06 | 0.85 | 1.32 | 0.6375 |  |  | 1.19 | 1.03 | 1.39 | 0.0223 |
| 3 |  | 1.51 | 1.11 | 1.86 | 0.0001 |  |  | 1.40 | 1.22 | 1.62 | <0.0001 |
| 4 |  | 1.55 | 1.55 | 2.04 | 0.0017 |  |  | 1.66 | 1.37 | 2.01 | <0.0001 |
| 1 | Middle-Aged (n=6,741) | 1.00 |  |  |  | Middle-Aged (n=6,734) | | 1.00 |  |  |  |
| 2 |  | 1.12 | 0.83 | 1.53 | 0.4634 |  |  | 1.30 | 1.09 | 1.56 | 0.0043 |
| 3 |  | 1.65 | 1.28 | 2.12 | 0.0001 |  |  | 1.46 | 1.25 | 1.72 | <0.0001 |
| 4 |  | 1.74 | 1.26 | 2.42 | 0.0009 |  |  | 1.66 | 1.34 | 2.05 | <0.0001 |
| 1 | Older Aged (n=2,397) | 1.00 |  |  |  | Older Aged (n=2,403) | | 1.00 |  |  |  |
| 2 |  | 1.02 | 0.74 | 1.41 | 0.9185 |  |  | 1.02 | 0.78 | 1.34 | 0.8914 |
| 3 |  | 1.19 | 0.81 | 1.75 | 0.3866 |  |  | 1.17 | 0.84 | 1.63 | 0.3531 |
| 4 |  | 1.23 | 0.73 | 2.05 | 0.4376 |  |  | 1.96 | 1.20 | 3.20 | 0.0076 |

*Each group of depressive symptoms is compared to the reference (Group 1)

**Table S5** **Main Effect Associations Between Social Isolation and CVDs (Complete Case Analysis)**

|  |  | | *HRD* | | | | | *PVRD* | | | | |
| --- | --- | --- | --- | --- | --- | --- | --- | --- | --- | --- | --- | --- |
| *Social Isolation (1 vs. 0)** | OR | 95% CI | | | P-value |  | OR | | | 95% CI | | P-value |
| Total  (n=5,555) | 0.75 | 0.30 | | 1.87 | 0.5316 | Total (n=5,553) | | | 2.42 | 1.18 | 4.97 | 0.0156 |
| Middle-Aged (n=4,182) | 1.64 | 0.56 | | 4.78 | 0.3648 | Middle-Aged (n=4,180) | | | 2.07 | 0.88 | 4.84 | 0.0941 |
| Older Aged (n=1,373) | 0.20 | 0.03 | | 1.60 | 0.1298 | Older Aged (n=1,373) | | | 4.21 | 0.92 | 19.21 | 0.0631 |

*Social isolation index is those who are socially isolated (coded as 1) compared to the reference not socially isolated (coded as 0)

**Table S6** **Interaction Associations Between PRS and Depressive Symptoms on CVDs (Complete Case Analysis)**

|  | | *HRD* | | | | | *PVRD* | | | |
| --- | --- | --- | --- | --- | --- | --- | --- | --- | --- | --- |
|  | PRS*Dep^+^ | OR | 95% CI | | P-value |  | OR | 95% CI | | P-value |
| Total (n=9,138) | PRS*1 | 1.00 |  |  |  | Total (n=9,137) | 1.00 |  |  |  |
|  | PRS*2 | 1.02 | 0.97 | 1.08 | 0.4645 |  | 0.97 | 0.93 | 1.00 | 0.0594 |
|  | PRS*3 | 0.99 | 0.95 | 1.05 | 0.8054 |  | 1.02 | 0.98 | 1.05 | 0.3273 |
|  | PRS*4 | 0.98 | 0.92 | 1.05 | 0.6368 |  | 1.00 | 0.95 | 1.04 | 0.8514 |
| Middle-Aged (n=6,741) | PRS*1 | 1.00 |  |  |  | Middle-Aged (n=6,734) | 1.00 |  |  |  |
|  | PRS*2 | 1.02 | 0.95 | 1.10 | 0.5515 |  | 0.97 | 0.93 | 1.02 | 0.2370 |
|  | PRS*3 | 1.00 | 0.94 | 1.06 | 0.8917 |  | 1.01 | 0.98 | 1.05 | 0.5215 |
|  | PRS*4 | 0.96 | 0.89 | 1.04 | 0.2822 |  | 1.00 | 0.95 | 1.06 | 0.9031 |
| Older Aged (n=2,397) | PRS*1 | 1.00 |  |  |  | Older Aged (n=2,403) | 1.00 |  |  |  |
|  | PRS*2 | 1.02 | 0.94 | 1.10 | 0.6424 |  | 0.95 | 0.89 | 1.01 | 0.1052 |
|  | PRS*3 | 1.00 | 0.91 | 1.09 | 0.9115 |  | 1.04 | 0.96 | 1.12 | 0.3618 |
|  | PRS*4 | 1.06 | 0.92 | 1.22 | 0.3967 |  | 0.97 | 0.86 | 1.09 | 0.5721 |

^+^Dep is the depressive symptoms group that is compared to reference (group 1)

**Table S7** **Interaction Associations Between PRS and Social Isolation on CVDs (Complete Case Analysis)**

|  | | | *HRD* | | | | | *PVRD* | | | |
| --- | --- | --- | --- | --- | --- | --- | --- | --- | --- | --- | --- |
|  | PRS*SII^+^ | OR | | 95% CI | | P-value |  | OR | 95% CI | | P-value |
| Total  (n=5,555) | PRS*1 | 0.96 | | 0.77 | 1.21 | 0.7566 | Total (n=5,553) | 1.12 | 0.93 | 1.35 | 0.2408 |
| Middle-Aged (n=4,182) | PRS*1 | 0.98 | | 0.74 | 1.30 | 0.8805 | Middle-Aged (n=4,180) | 1.19 | 0.88 | 1.43 | 0.1656 |
| Older Aged (n=1,373) | PRS*1 | 1.04 | | 0.66 | 1.63 | 0.8766 | Older Aged (n=1,373) | 1.03 | 0.74 | 1.44 | 0.8669 |

^+^SII is those who are socially isolated (coded as 1) compared to the reference not socially isolated (coded as 0)

**Table S8** **Main Effect and Interactive Associations Between PRS and Social Isolation on CVDs (More Conservative Statistical Model)**

|  | |  | *HRD* | | | *PVRD* | | | | |
| --- | --- | --- | --- | --- | --- | --- | --- | --- | --- | --- |
| Exposure | Group | OR | 95% CI | | P-value | | OR | 95% CI | | P-value |
| PRS |  |  |  |  |  | |  |  |  |  |
|  | Total | 1.06 | 1.05 | 1.07 | <0.0001 | | 1.01 | 1.00 | 1.02 | 0.0168 |
|  | Middle-Aged | 1.06 | 1.03 | 1.08 | <0.0001 | | 1.01 | 1.00 | 1.03 | 0.0453 |
|  | Older-aged | 1.06 | 1.04 | 1.09 | <0.0001 | | 1.01 | 1.00 | 1.03 | 0.3570 |
| Depressive Symptoms* |  |  |  |  |  | |  |  |  |  |
| 1 | Total | 1.00 |  |  |  | | 1.00 |  |  |  |
| 2 |  | 1.06 | 0.85 | 1.32 | 0.6375 | | 1.10 | 0.94 | 1.28 | 0.2497 |
| 3 |  | 1.51 | 1.11 | 1.86 | 0.0001 | | 1.26 | 1.09 | 1.46 | 0.0018 |
| 4 |  | 1.55 | 1.55 | 2.04 | 0.0017 | | 1.28 | 1.05 | 1.56 | 0.0139 |
| 1 | Middle-Aged | 1.00 |  |  |  | | 1.00 |  |  |  |
| 2 |  | 1.12 | 0.83 | 1.53 | 0.4634 | | 1.11 | 0.92 | 1.34 | 0.2689 |
| 3 |  | 1.65 | 1.28 | 2.12 | 0.0001 | | 1.30 | 1.11 | 1.53 | 0.0016 |
| 4 |  | 1.74 | 1.26 | 2.42 | 0.0009 | | 1.28 | 1.03 | 1.60 | 0.0288 |
| 1 | Older Aged | 1.00 |  |  |  | | 1.00 |  |  |  |
| 2 |  | 1.02 | 0.74 | 1.41 | 0.9185 | | 1.08 | 0.83 | 1.40 | 0.5835 |
| 3 |  | 1.19 | 0.81 | 1.75 | 0.3866 | | 1.13 | 0.82 | 1.57 | 0.4496 |
| 4 |  | 1.23 | 0.73 | 2.05 | 0.4376 | | 1.45 | 0.91 | 2.30 | 0.1164 |
| SII^+^ |  |  |  |  |  | |  |  |  |  |
|  | Total | 0.92 | 0.53 | 1.60 | 0.7810 | | 1.86 | 1.20 | 2.89 | 0.0057 |
|  | Middle-aged | 1.59 | 0.78 | 3.22 | 0.2000 | | 1.99 | 1.11 | 3.58 | 0.0214 |
|  | Older-aged | 0.51 | 0.22 | 1.17 | 0.1129 | | 1.64 | 0.81 | 3.31 | 0.1707 |
| PRS*  Depressive symptoms^†^ |  |  |  |  |  | |  |  |  |  |
| PRS*Depr 1 vs. 4 | Total | 1.03 | 0.99 | 1.09 | 0.1938 | | 0.98 | 0.94 | 1.01 | 0.2386 |
| PRS*Depr 2 vs. 4 | Total | 0.99 | 0.94 | 1.04 | 0.7311 | | 1.01 | 0.98 | 1.05 | 0.5005 |
| PRS*Depr 3 vs. 4 | Total | 1.04 | 0.94 | 1.06 | 0.4889 | | 0.99 | 0.94 | 1.03 | 0.5388 |
| PRS*Social isolation |  |  |  |  |  | |  |  |  |  |
| PRS*SII^†^ | Total | 1.08 | 0.94 | 1.23 | 0.2890 | | 1.10 | 0.98 | 1.24 | 0.1024 |

Model adjusted for age, the first five principal components of ancestry, biological sex, education level, province at recruitment, total household income, smoking status, urban/rural classification, immigration status, waist circumference, total cholesterol:HDL cholesterol (ratio), physician-diagnosed diabetes, physical activity level, alcohol intake, fruit and vegetable intake, and whole grains intake.

*Each group of depressive symptoms is compared to the reference (Group 1).

^+^SII is those who are socially isolated (coded as 1) compared to the reference not socially isolated (coded as 0).

^†^No significant interactions were observed in the stratified age group analysis (data not shown).
